# Assessment of Seasonal Variation in Methane Emissions of Mediterranean Buffaloes Using a Laser Methane Detector

**DOI:** 10.3390/ani12243487

**Published:** 2022-12-09

**Authors:** Lydia Lanzoni, Mizeck G. G. Chagunda, Isa Fusaro, Matteo Chincarini, Melania Giammarco, Alberto Stanislao Atzori, Michele Podaliri, Giorgio Vignola

**Affiliations:** 1 Facoltà di Medicina Veterinaria, Università degli Studi di Teramo, Piano d’Accio, 64100 Teramo, Italy; 2 Animal Breeding and Husbandry in the Tropics and Subtropics, University of Hohenheim, 70599 Stuttgart, Germany; 3 Dipartimento di Agraria, Sezione di Scienze Zootecniche, University of Sassari, Viale Italia 39, 07100 Sassari, Italy; 4 Istituto Zooprofilattico Sperimentale dell’Abruzzo e del Molise, Campo Boario, 64100 Teramo, Italy

**Keywords:** sustainability, greenhouse gas emissions, methane, buffaloes, non-invasive tool, heat stress

## Abstract

**Simple Summary:**

Methane is a powerful greenhouse gas also released by ruminants due to their physiological digestive process. A strong knowledge of the emission level of each species and on the variability, factors is needed to target the best mitigation strategy for the livestock sector. To assess these emissions, non-invasive and feasible technologies, such as Laser Methane Detectors (LMD), are now available. The present study represents the first assessment of methane emissions in Italian Mediterranean buffaloes with LMD and investigates the effect of the season on these emissions. The results obtained showed that the season significantly influenced emissions, since lower values were found in summer when compared to the winter period; therefore, this should be considered when setting up a measurement protocol with LMD.

**Abstract:**

A direct assessment of the methane (CH_4_) emission level and its variability factors is needed in each animal species in order to target the best mitigation strategy for the livestock sector. Therefore, the present study aimed to (1) test a laser methane detector (LMD) for the first time in Italian Mediterranean buffaloes (IMB), a non-invasive tool to quantify CH_4_ emissions; (2) test the effect of season on the emissions; and (3) compare the results measured directly with the ones estimated with the existing equations. CH_4_ emissions of twenty non-productive IMB, under the same feeding regimen, were monitored for 12 days in summer and winter. Significantly higher THI (74.46 ± 1.88 vs. 49.62 ± 4.87; *p* < 0.001), lower DMI (2.24 ± 0.04 vs. 2.51 ± 0.03% DMI/kg live weight; *p* < 0.001) and lower emission intensities (0.61 ± 0.15 vs. 0.75 ± 0.13; *p* < 0.001) were found during the summer period when compared with winter. LMD was found to be a versatile tool to be used in buffaloes, and it was clear that a summer increase in THI could act as a stressor for the animals, influencing their emissions. In addition, measured emissions were significantly higher than when estimated with the existing equations (*p* < 0.001), suggesting the need for further research in this area.

## 1. Introduction

In Southern Italy, Mediterranean buffaloes are mainly farmed for milk production to be used for dairy processing into “mozzarella” cheese. The growing interest in the mozzarella cheese induced a growth in the number of farmed dairy buffaloes, which in Italy increased from approximately 100,000 in the 1980s to over 400,000 in 2021 [1,2].

In ruminants, methane (CH_4_) production is part of the physiological digestive process through enteric fermentation and contributes to global warming. In fact, CH_4_ is a powerful greenhouse gas with a high global warming potential, both if evaluated with the GWP_100_ (global warming potential in a 100-year period) [3,4] and with the recent GWP* approach [5]. In November 2021, at the United Nations Climate Change Conference (COP-26), “The Global Methane Pledge” was signed, which commits countries involved to a reduction of 30% in methane emissions deriving from anthropogenic activities [6]. Among the pledge’s objectives for the agricultural sector, together with the Climate and Clean Air Coalition, is the reduction of methane from animal’s enteric fermentation per unit of product [7]. 

Therefore, given the increasing importance of the buffalo farming industry, evidence-based data are essential to increase the knowledge of the CH_4_ emission level of this species and to refine breed- and country-specific emission factors to support a more accurate inventory in order to improve the accuracy of IPCC equations for each animal category (i.e., growing, lactating or dry) [8]. In this respect, [9] reported a consistent contribution of growing buffaloes to the overall methane emission of this species.

Metabolic chambers are the “gold standard” for assessing emissions, since they are the most precise and accurate method and provide a controlled environment at the experimental level, although they are expensive, time-consuming, not flexible for on-farm monitoring and the confinement of the animals in the chamber might affect their behaviour and welfare [10,11]. The sulphur hexafluoride (SF_6_) tracer technique is suitable for measuring methane emissions while maintaining animals in their natural conditions (i.e., grazing), although it is labour-intensive and invasive for the animals since they require the placement of a permeation tube and a bolus with a known SF_6_ gas released into the reticulorumen [12]. Other methods have been developed that require a short measurement period and that can be used on-farm to record CH_4_ emissions in a non-invasive way, such as the GreenFeed [13], breath analysers installed in the feeding bin or in the milking systems [14,15,16] and laser-based methods such as laser methane detectors (LMD). 

An LMD is a handheld, portable smart tool developed for the detection of gas leaks from a safe distance in gas transmission networks, landfills and other areas. Its use in livestock was first reported by [17], and from there it has been widely applied to several species such as dairy cows [17,18,19,20,21,22,23], beef cattle [24], sheep [24,25] and goats [26,27]. An LMD uses a high selectivity infrared absorption spectroscopy method to detect the CH_4_ concentration in the breath of animals. It uses a semiconductor laser as a collimated excitation source and employs the second harmonic detection of wavelength-modulation spectroscopy for the measurement. A guiding laser in the visible spectrum helps to direct the invisible measuring laser to the desired target [19,28]. The CH_4_ concentration between the detector and the target is measured by assessing a fraction of the diffusely reflected laser beam [29]. Infrared spectroscopy techniques, although they have only recently started to be adopted for breath analysis, appear promising since they are less time-consuming than traditional laboratory techniques [19,30]. The possibility of obtaining measurements in real time has important advantages, especially when dealing with animals, as measurements must be made quickly and safely [19]. 

To date, no studies are available concerning direct measurement of CH_4_ emissions from Mediterranean buffaloes, while a few authors measured CH_4_ emissions from buffaloes of Murrah and Bhadawari breeds with the SF6 tracer technique [31,32,33,34,35,36]. Moreover, LMD was never used as a tool to measure CH_4_ emissions in any of the aforementioned buffalo breeds. Despite the lower accuracy of LMD, when compared with other methods, it represents a flexible and non-invasive technique to compare emissions among different situations [28]. 

Many factors can influence CH_4_ emissions, including the thermal comfort of the animal, which is affected by the temperature-humidity index (THI) and can influence the animal’s behaviours and dry matter intake [37,38].

Therefore, the aim of the present study was to (1) test the feasibility of using breath as a medium for quantifying CH_4_ emissions in Mediterranean buffaloes with an LMD; (2) explore methodological aspects related to the LMD assessment protocol; (3) provide evidence-based on-farm collected data on the CH_4_ emission of growing buffaloes and compare them with the existing equations of the Intergovernmental Panel on Climate Change (IPCC, 2019); and (4) evaluate the effect of the season on the CH_4_ emissions of the animals.

## 2. Materials and Methods

### 2.1. Animals and Housing

The study was conducted at the experimental farm of the “Istituto Zooprofilattico Sperimentale dell’Abruzzo e del Molise” (Teramo, Italy, 42°41′25.0″ N 13°44′25.1″ E) during two study periods in 2021: four weeks in summer (July) and four weeks in winter (December). In total, 20 growing Italian Mediterranean buffaloes (11 females and 9 males), housed at the experimental farm for other research purposes, were enrolled in the study. The animals were homogeneous in age (18 ± 1.5 months at the beginning of the trial), not pregnant, and not lactating. The animals were group-housed together in the stalls, with access during the morning and early afternoon to an outdoor paddock adjacent to the barn, which was provided with a natural waterhole for wallowing. The stable was equipped with straw bedding that was changed weekly.

### 2.2. Diet 

The animals were fed with ad libitum mixed hay and 3 kg/head/day of commercial concentrate feed, administered twice a day (9 a.m. and 3 p.m.). Water was provided ad libitum in eight linear drinking troughs. During the trial, samples of the hay and concentrate were collected every two weeks and analysed for dry matter content (DM), ash and crude protein (CP) following the official methods of [39]. Neutral detergent fibre (NDF), acid detergent fibre (ADF) and lignin (ADL) were analysed as described by [40]. The results of the feed analysis are reported in Table 1.

The dry matter intake was estimated twice a week for both the summer and winter periods based on the group’s forage and concentrate consumption. This value was then divided by the number of animals in the barn to obtain the individual average DMI and expressed as % of the live weight of the animal (LW). The LW used for this calculation was the average weight of the animals, measured in each trial season. 

### 2.3. Animals’ Weight Estimation 

It was not possible to perform a weighing of the animals; hence, an estimation was performed using two different equations: (1) by Riaz et al., 2018 [41] and (2) by Khan et al., 1978 [42], with a measuring tape.

(1) $$\mathrm{Body}\;\mathrm{Weight}\;\left(\mathrm{kg}\right)=\frac{{\left(\mathrm{HG}\right)}^{2}\times \;\mathrm{DBL}}{300}\times 0.453592$$

(2) $$\mathrm{Body}\;\mathrm{Weight}\;\left(\mathrm{kg}\right)=1697.226+\left(16.761\times \;\mathrm{height}\right)+\left(23.947\times \;\mathrm{HG}\right)+\;\left(0.514\times \;\mathrm{DBL}\right)\times 0.453592\;$$

where height indicates height at withers, HG is heart girth and DBL is diagonal body length. All expressed in inches in the equations. Both equations were multiplied by 0.453592 to convert libras into kilograms.

The heart girth was measured using a measuring tape, drawn from a point slightly behind the shoulder blade down to the sixth rib position and under the body behind the elbow [41]. The diagonal body length was taken from the point of the shoulder to the point of the pin bone and the height of the animals was measured at withers.

### 2.4. Temperature and Humidity Index (THI) Measurements

Three temperature and humidity data loggers (RHT30 model, Extech, Nashua, NH, USA) were placed at the same level as the animals inside the barn, and had a sampling frequency of 10 min during the whole duration of the study. The average of the environmental temperature and relative humidity from the three data loggers was used to calculate the temperature-humidity index (THI) of the 24 h, using the NRC equation (1971) [43]:

$$\mathrm{THI}=\left(1.8\times \;T+32\right)-\left[\left(0.55-0.0055\times \;\%\mathrm{HR}\right)\times \left(1.8\times \;T-26\right)\right]$$

where T is environmental temperature in °C and %HR is relative humidity.

### 2.5. CH_4_ Emissions Evaluation

Methane measurements were performed on three days for four consecutive weeks both in the summer and winter period using a hand-held laser methane detector, “Laser Methane mini™ (LMm)” model (Tokyo Gas Engineering Co., Ltd., Tokyo, Japan). In the first period, measurements were performed in the morning (10:00–12:30), while in the second period, measurements were performed in the morning (10:00–12:30) and evening (16:00–18:30), to evaluate the effect of the time of the day on methane emissions. The measurements were obtained at least 1 h after concentrate feeding since previous studies demonstrated how this variable can influence the measurement [24,44]. The measurements were carried out by the same trained operator pointing the green laser beam of the laser methane detector (LMD) at the nostrils of the buffaloes. The LMD measures the concentration (ppm × m) of CH_4_ present between the animal and the detector with the principle of infrared spectroscopy with a wavelength set on high selectivity for the band of CH_4_ absorption. The LMm operates in a temperature range between −17 °C and +50 °C and a humidity range of 30–90%. It can detect CH_4_ concentrations between 1 and 50,000 ppm × m with a detection accuracy of ±10% and can operate from a distance up to 30 m from the emission source. For all measurements, a fixed distance of one meter was maintained between the buffalo’s muzzle and the device. The distance was controlled using a laser distance meter (DISTO D2 model, LEICA, Heerbrugg, Switzerland); at least six distance measurements were performed during the monitoring of each animal. The measurement was performed with the animals standing idle in a corridor without physical restraint. Measurements were performed with a continuous recording every 0.5 s for a 4-min duration to capture the full eructation cycle, as suggested by previous studies [17,19,45] and confirmed in the recent research by [46]. LMD was connected to a tablet running the GasViewer app via a Bluetooth connection for exporting and storing the data. At the beginning of each measurement session, CH_4_ emissions from bedding, measured at one meter of distance, were evaluated from three different spots and their average was calculated.

The output of the LMD recording consists of a time series of values of CH_4_ emissions belonging to a single animal that consists of peaks and troughs representing the inhalation and exhalation of the respiratory cycle. When an eructation event occurs, the individual peaks are much higher, therefore it is necessary to divide them from the respiratory values [17,23,26,28,45]. Mean and standard deviation (s.d.) were calculated for each profile, and one s.d. was used as a cut-off value to discriminate between methane emission from breathing (CH_4_ breath) and eructation (CH_4_ peak) as described by [45]. The averages of the CH_4_ peak, CH_4_ breath and of the overall CH_4_ concentrations (CH_4_ average), were used to perform further analysis. Thereafter, data were converted from ppm–m to kg/year with the equation developed by [17,47] that estimates CH_4_ emission from the tidal respiratory volume as follows:

$${\mathrm{CH}}_{4\;}\left(\frac{g}{\min}\right)={\mathrm{CH}}_{4\;}\mathrm{peak}\;\times \;V\;\times \;R\;\times \;\alpha \times \;\beta \;\times 10^{-6}$$

$${\mathrm{CH}}_{4\;}\left(\frac{g}{\mathrm{day}}\right)={\mathrm{CH}}_{4\;}\left(\frac{g}{\min}\right)\times 1440$$

$${\mathrm{CH}}_{4\;}\left(\frac{\mathrm{kg}}{\mathrm{year}}\right)={\mathrm{CH}}_{4\;}\left(\frac{g}{\mathrm{day}}\right)\times \frac{365}{1000}$$

where g is grams; CH_4_ peak is the average of the peak value of emission in ppm–m divided by the distance from the animal (1 m); V is tidal volume, determined as 3800 mL; R is respiratory rate, determined as 20 acts per minutes; α is conversion factor of methane production from mL to gram, which is 0.000667 g/mL; and β is dilution factor, which is ten, to correct for the difference between breath and total methane production.

Thereafter, the emission intensity was calculated and expressed as gCH_4_/day/kg LW (live weight) by dividing the daily CH_4_ emissions in g/day by the average weight of the animals.

Moreover, to allow a comparison, Tier 2 method was applied to calculate the methane emissions from individual animals for the two seasons, estimating the DMI as % of LW of the animals, using the following (IPCC, 2019) equation:

$$\mathrm{GEI}\left(\frac{\mathrm{MJ}}{\mathrm{day}}\right)=\mathrm{DMI}\;\times 18.45\;$$

$${\mathrm{CH}}_{4}\left(\frac{\mathrm{kg}}{\mathrm{year}}\right)=\frac{\mathrm{GEI}\times \left(\frac{Y_{m}}{100}\right)\times 365}{55.65}\;$$

where GEI is gross energy intake; DMI is dry matter intake, estimated as % of the live weight of the animal; 18.45 is conversion factor for dietary GE per kg of dry matter; and Y_m_ is methane conversion factor, or the % of GE in feed converted to methane. The value used was 6%, as reported by [48] for sub-adult Mediterranean buffaloes (1–3 years old); 55.65 indicates energy content of methane in MJ/kg CH_4._

### 2.6. Statistical Analysis 

Statistical analyses were performed in R version 4.0.4, via the R studio version [49]. Overall and seasonal descriptive statistics are expressed in the paper as mean ± s.d.

Linear models were used to test the effect of season on the DMI (% of LW) and on THI. The correlation between weight estimated with the two different equations was performed with the “rmcorr” package [50], used for repeated measurement. Since a high correlation (r = 0.9) was found between equations, the average weight value was used to test the seasonal differences (ID as a random effect) and those related to the sex of the animals (ID and season as a random effect) using a linear mixed model.

Linear models were used to test the differences in CH_4_ emissions from bedding between days and to test their effect on CH_4_ average, CH_4_ peak and CH_4_ breath. To test the effect of the time of the recording (morning vs. evening) performed in winter on the CH_4_ average, CH_4_ peak and CH_4_ breath, data were analysed with a mixed model, using the ID of the animals and the day of the recording as a random effect. Since no significant differences were found, a daily average was used to perform further analyses. 

Linear mixed models were finally used to assess the effect of LW on CH_4_ peak, breath and average emissions measured with LMDs, and of the season on the emission intensity (EI), retaining the animal’s ID as a random effect. In addition, a linear mixed model was also used to compare kgCH_4_/head/year from LMD recording with the individual values estimated with IPCC equations, retaining the animals’ ID as a random effect. Values were considered significant when *p* < 0.05.

## 3. Results

This section may be divided by subheadings. It should provide a concise and precise description of the experimental results, their interpretation as well as the experimental conclusions that can be drawn.

### 3.1. Seasonal Weight, DMI and THI 

A strong positive correlation was found between the weight of the animals calculated with the equation by [41] and by [42] (r = 0.99; *p* < 0.001)l; therefore, the average values of the results obtained with the two estimations were used to conduct further analyses. A significant increase in the body weight of the animals was observed from the summer (398.3 ± 41.4 kg; 18 ± 1.5 months of age) to the winter period (497.3 ± 47.3 kg; *p* < 0.001; 23 ± 1.5 months of age), while no significant differences were found between the sexes. 

The average percentage of dry matter intake on the animal’s body weight was found to be significantly lower during summer (2.24 ± 0.04% DMI/kg live weight) than during winter (2.51 ± 0.03% DMI/kg live weight; *p* < 0.001). 

**Figure 1:**
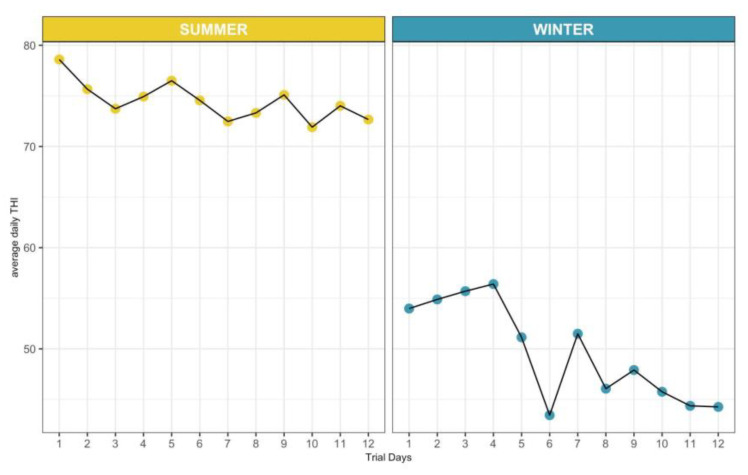
Variation of the average temperature and humidity index in the 12 summer and winter trial days.

In addition, the average daily THI was found to be significantly lower during winter trial days (summer: 74.46 ± 1.88; winter: 49.62 ± 4.87; *p* < 0.001), while no differences between the different days within the seasons were observed (Figure 1).

### 3.2. CH_4_ Emissions Evaluation with LMD

There was no significant variation in CH_4_ concentrations in the bedding over the trial days (14.18 ± 4.83 ppm–m). This was the case in both the summer and the winter period. Moreover, no influence of CH_4_ bedding was found on CH_4_ average, CH_4_ peak and CH_4_ breath; therefore, those “offset” values were not subtracted from the methane emission data. 

During the winter period, no significant differences were noted among the evaluations performed at the different times of the day for CH_4_ average (morning = 39.8 ± 12.9; evening = 41.4 ± 11.7), CH_4_ peak (morning = 125.9 ± 56.9; evening = 134.8 ± 55.0) and CH_4_ breath (morning = 21.2 ± 7.0; evening = 22.0 ± 5.9); therefore, the winter data were averaged for each day of the assessment. 

The overall mean, including both seasons, of CH_4_ peak, CH_4_ breath and CH_4_ average measured with LMD was found to be 115.8 ± 56.4 ppm–m, 18.3 ± 7.4 ppm–m and 34.2 ± 14.3 ppm–m, respectively as reported in Table 2. When converting the CH_4_ peak results from ppm-m into g/day and kg/year the overall mean values of methane emission were found to be 328.6 ± 160.0 g/day and 119.9 ± 58.4 kg/year.

No significant effect of the animal’s body weight was found on CH_4_ peak, breath and average measured with LMD. To assess the seasonal differences in methane emissions, since the animals were growing, data were expressed as emission intensity (EI, g CH_4_/day/kg LW), and a significantly higher EI was observed during the winter period (*p* < 0.001; Table 3). 

### 3.3. CH_4_ Emissions Estimated with IPCC Equations

**Figure 2:**
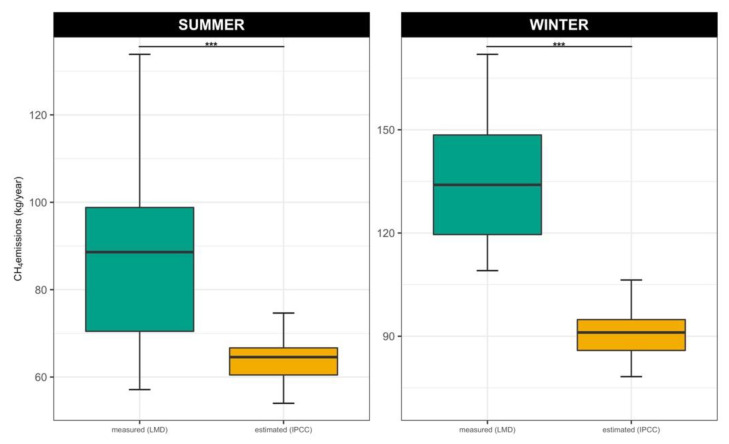
Seasonal CH_4_ average values (kg/head/year) estimated with IPCC equation or measured with LMD (*** = *p* < 0.01 between estimated and measured values).

The results obtained with the Tier 2 approach suggested by IPCC 2019 are shown in Figure 2. Significantly lower values were found when estimating methane emissions in kg/head/year with the IPCC equations than when recording with LMD (*p* < 0.001).

## 4. Discussion

The study presents original results on the assessment of methane emissions in growing Mediterranean buffaloes through the monitoring of the breath using the LMD and proposes an overview of their seasonal variations. LMD was confirmed to be a versatile tool [28,45], easily usable on buffaloes that allows on-farm monitoring in a non-invasive way for the animals. 

Regarding the methodological aspects of the LMD assessment protocol, different authors accounted for methane concentrations from bedding or from the environment as a background value that was subtracted from the recording on animals [22,27]. However, from the results of the present study, CH_4_ from bedding did not influence the recording of the animals when monitoring them from a one-meter distance, as shown by [23]. 

While [27] described a consistent diurnal pattern in CH_4_ emissions, the results of the present study collected during the winter period to compare the morning and evening emissions did not demonstrate this difference. Refs. [24,44] demonstrated an effect on CH_4_ diurnal variation of the distance between feeding and the measurement. Therefore, the absence of differences found herein was probably due to the observance of a fixed amount of elapsed time of 1 h between feeding and recording, both for the morning and evening evaluation. These methodological results can provide an interesting outcome to improve LMD monitoring guidelines for a uniform measurement and data-analysis protocol [28,46].

To our knowledge, there are no studies in the literature where methane emissions are evaluated with LMDs on buffaloes; thus, the comparison can only be performed with data collected from dairy and beef cattle. This comparison should be interpreted with caution, considering the differences in emissions associated with species-specific factors, age, size, feeding regimen and the productive state of the animals [51]. 

The overall CH_4_ peak concentration found in the present study (115.8 ± 56.4 ppm–m) was comparable with the results obtained by [23] (129 ppm–m) and [18] (114 ppm–m) in dairy cows and by [24] in beef cattle fed with a forage-based diet (110.7 ± 4.91 ppm–m). Higher values were reported by [17,19] (201 ppm–m and 396 ppm–m, respectively) in dairy cows; this difference could be attributable to the methods used for the LMD data handling performed in the latter studies, i.e., using two standard deviations (instead of one, as used in the present study) to discriminate CH_4_ peak emissions associated with eructation. 

Concerning the measurement of methane emissions in buffaloes, some authors performed the assessment in different breeds with the sulphur hexafluoride (SF_6_) tracer technique, which was later converted into gCH_4_/head/day. Most of these studies, as can be seen in Table 4, involved Murrah buffaloes of different ages and weights, with a high variation in the methane emission measured (from 59 to 301 gCH_4_/head/day), mostly related to the differences in breed, animal weight, DMI and in the diet used. In particular, the results found in the present study (328.6 ± 160.0 gCH_4_/head/day) are comparable with the results obtained from animals of similar body weight and DMI ([35] for Murrah buffaloes). In this respect, a positive correlation between DMI and CH_4_ emissions in ruminants is already well-established [44,52,53,54]. 

On the contrary, the present study did not show an effect of an animal’s weight on CH_4_ emissions, differently from the findings of [38,55] in cattle, but in agreement with the results of [56], that showed how body weight was not a good CH_4_ emission predictor for sheep. Further studies should, then, be performed to establish if body weight should be included as a good CH_4_ emission predictor for Mediterranean buffaloes. 

In order to compare CH_4_ emissions between the summer and winter season, EI (gCH_4_ day/kg of live weight) was used to normalise the values, because the animals were growing. As in the present study, a significantly higher emission value was observed during winter by [38,57] in bovines and by [58] in buffaloes. On the contrary, [36] did not find any influence of the season on CH_4_ emissions. According to [57], the increase in environmental temperature during summer causes a reduction in animal activity, which seems to correlate with a reduction in methane emission (r = 0.66). [58] attributed lower values of emissions during the heat stress period to a decrease in thyroid activity, which could be reflected in a reduction in gastrointestinal motility and in a consequent increase in transit time [59]. In addition, [38] showed how animals under heat stress reduce DMI and, consequently, their methane emissions. Considering the classification of [60] for Mediterranean buffaloes, animals start experiencing mild heat stress with a THI ranging from 72 to 78. Therefore, the animals involved in the present study were found to be subjected to mild heat stress for the whole duration of the summer recordings, which can explain the reduction in DMI [61]. Thus, the most important cause of EI reduction during the summer period observed in the present study could be associated with the significant decrease in DMI when compared with the winter period. 

The significant difference found comparing our results with those obtained with the IPCC equations for the estimation of CH_4_ enteric emissions could suggest the need for an improvement for species-specific and possibly season-specific research-based data to implement the currently available emission coefficient for Mediterranean sub-adult buffaloes [48]. 

However, it should be noted that the present study was conducted on a relatively low number of non-lactating animals, monitored for a short period of time, and the methane emission was assessed only at a specific time of the day with an LMD. In addition, since an LMD measures concentration and gas flux, lower accuracy and precision was evidenced by [28], when compared with other methods tested to monitor direct CH_4_ from animals. Therefore, it could have limitations in fully representing the fluctuations of methane emissions, and results should be confirmed by further studies.

## 5. Conclusions

In conclusion, the following study reports, for the first time, data on the CH_4_ emission of Mediterranean buffaloes obtained directly on-farm with a non-invasive tool such as an LMD. For this species, this device has proven to be a versatile and easy-to-use tool which can be adopted in the future to measure emissions in buffaloes under different productive stages. 

No interference of the bedding emissions was found on methane recording and no differences in diurnal monitoring were shown if the assessment was performed at a constant interval from feeding, allowing for the standardisation of the existing protocols for these aspects. Despite the lower precision and accuracy level of an LMD when compared with other methods, it was confirmed to be a suitable tool to compare the emissions of the animals during two different seasons. The seasonal emission assessment, associated with changes in THI, was confirmed as a relevant variable reflected in animal DMI and CH_4_ emissions. Further studies will be required to deepen the predominant mechanism associated with these variations and to investigate more precisely the effect of heat waves and/or cold spells. In addition, further studies should be addressed at assessing additional sources of emission variations in buffaloes to obtain a more comprehensive overall picture. The differences found in the comparison between the CH_4_ emissions measured and estimated through the IPCC equations suggest that future research should focus on deepening and refining the existing models for Mediterranean buffaloes from data collected directly on-field. The development of a user-friendly method to monitor emissions and a thorough knowledge of all associated influencing variables could help identify mitigation strategies to reduce emissions from buffaloes and, consequently, from the livestock sector.

## Figures and Tables

**Table 1:** Chemical composition of concentrate and hay (% on a DM basis) used during the trial.

Chemical Composition	Concentrate	Hay
DM	89.73	91.47
CP	18.25	13.90
NDF	25.84	55.10
ADF	9.45	41.16
ADL	2.75	9.18
Ash	7.40	7.40

CP = crude protein; NDF = neutral detergent fibre; ADF = acid detergent fibre; ADL = acid detergent lignin.

**Table 2:** Overall average values recorded during the trial, expressed as mean ± s.d.

Variables	Value
Weight, kg	429.9 ± 65.8
DMI (% of LW)	2.38 ± 0.14
Methane peak, ppm–m	115.8 ± 56.4
Methane breath, ppm–m	18.3 ± 7.4
Methane average, ppm–m	34.2 ± 14.3
Daily methane emission, gCH_4_/day	328.6 ± 160.0
Yearly methane emissions, kgCH_4_/year	119.9 ± 58.4

**Table 3:** Seasonal average values (summer and winter) recorded during the trial, expressed as mean ± s.d.

Measure	Summer	Winter
Weight, kg	398.3 ± 41.4 ^B^	497.3 ± 47.3 ^A^
DMI, % LW	2.24 ± 0.04 ^B^	2.51 ± 0.03 ^A^
Emission Intensity, gCH_4_/day/live weight	0.61 ± 0.15 ^B^	0.75 ± 0.13 ^A^

Means with different superscript (^A, B^) letters were found statistically different with *p* < 0.001.

**Table 4:** Studies on direct CH_4_ emissions measured with sulphur hexafluoride (SF_6_) tracer technique on buffaloes.

Author	Breed	Weight (kg)	DMI (%LW)	F:C	CH_4_ (g/day)
[31]	Murrah	135.0	3.00	60:40	59
[32]	Surti × Mehasana	503.4	3.05	70:30 (Control)	257
		509.9	3.02	80:20 (Experimental)	230
[33]	Murrah	284.0	2.42	70:30	93
[34]	Murrah	218.5	1.95	100:0	70–78
[35]	Bhadawari	441.9	2.03	57:43	183
	Murrah	515.4	2.38	66:34	301
[36]	Murrah	158.5	2.35 (S)	65:35 (Diet 1)	90–100
			2.49 (W)		
			2.40 (S)	55:45 (Diet 2)	80–90
			2.58 (W)		
			2.21 (S)	70:30 (Diet 3)	100–115
			2.49 (W)		

Weight and DMI are expressed as mean values. F:C = forage to concentrate ratio. S = summer; W = winter.

## Data Availability

The data presented in this study are available on request from the corresponding author.
